# The Effects of Social Support on Strenuous Physical Exercise

**DOI:** 10.1007/s40750-017-0086-8

**Published:** 2018-01-11

**Authors:** Arran Davis, Emma Cohen

**Affiliations:** 1 Institute of Cognitive and Evolutionary Anthropology, University of Oxford, 64 Banbury Road, Oxford, OX2 6PN UK; 2 Wadham College, Parks Road, Oxford, OX1 3PN UK

**Keywords:** Social support, Exercise, Self-regulation, Performance-enhancement, Endogenous analgesia

## Abstract

In humans, socio-environmental cues play an important role in determining adaptive psychophysiological states and behaviours. In sport and exercise, cues to cohesive groups and close, supportive relationships are ubiquitous, possibly because of their effects on neurobiological mechanisms underlying physical performance. Clinical research has shown that the presence of supportive others can lead to reductions in perceptions of pain, while research from sport and exercise science has shown that pain and physical fatigue occupy ranges on a single spectrum of physical discomfort, which works to regulate outputs during strenuous physical exertion. Given the similar neurobiological underpinnings of pain and fatigue, the involvement of both in the self-regulation of strenuous physical outputs, and the effects of social support on perceptions of pain and neurophysiological stress responses more generally, we hypothesised that perceptions of social support affect outputs during strenuous physical exercise by altering activity in self-regulatory mechanisms involved in perceptions of pain and fatigue. We used a between-subjects experimental design to test this hypothesis, varying participants’ social support and measuring physical outputs and perceptions of physical discomfort and exertion during a series of maximum-effort cycling bouts. Analyses showed that participants in the social support condition produced greater initial outputs and steeper declines over time, compared to controls. This effect was moderated by participant neuroticism; an important predictor of how individuals react to social support. We discuss these findings in terms of proposed causal mechanisms linking supportive, cohesive social environments with self-regulation and physical performance.

**Electronic supplementary material:**

The online version of this article (10.1007/s40750-017-0086-8) contains supplementary material, which is available to authorized users.

## Introduction

Studies across a variety of fields using ethnographic, observational, and experimental methods suggest that team cohesion and the presence of close, supportive relationships can enhance performance during sport and exercise via reductions in physical discomfort and fatigue (Davis et al. 2015; Gotaas 2012; Kraus et al. 2010). However, the effects of the presence of close supportive others – an operationalised form of emotional social support (Heinrichs et al. 2003; Schnall et al. 2008) – on physical performance have not been tested experimentally, leaving it an open question as to whether and how social support affects pain and fatigue and the physical outputs they govern. This study tested the hypothesis that social support influences everyday exercisers’ performance during anaerobic exertion by altering activity in self-regulatory mechanisms involved in perceptions of pain and fatigue.

In social species, for which survival and reproduction correlate positively with close, stable relationships with conspecifics, social environments can have strong psychophysiological influences (Kikusui et al. 2006). Research has shown that the safety signalled by the presence of conspecifics, and especially bonded conspecifics, can ‘buffer’ adverse responses to threatening stimuli, as measured, for example, by reductions in indicators of stress, fear, and pain (Kiyokawa et al. 2004; Kikusui et al. 2006). In humans, where the presence of close social relationships is correlated not only with safety, but also care and resource abundance, social support is associated with a diverse range of positive psychophysiological effects (Hennessy et al. 2009). Perceptions of both situational and daily levels of social support can lead to reductions in stress responses and perceived pain unpleasantness, as well as improvements in healing and immunity (Cohen et al. 2015; Roberts et al. 2015; Eisenberger et al. 2011; Kulik and Mahler 1989). The presence of social support thus seems to change the parameters of adaptive behaviour in response to a range of stressors, threats, and risks.

Evidence suggests that social support acts as a psychosocial resource that reduces recipients’ appraisals of threat or impending adversity in physical and social challenges, and this can have downstream effects on a variety of psychophysiological responses (Lazarus and Folkman 1984). Physical exertion incurs costs in resource use, and it risks possible exhaustion and injury. Mechanisms modulating pain, fatigue, and predictions and perceptions of difficulty in physical challenges are thought to result from selection for adaptive exertion levels given specific and variable configurations of internal and external environments (Noakes et al. 2005; Noakes 2012; Humphrey and Skoyles 2012). While pain, fatigue, and heightened perceptions of physical challenge can help to protect against overexertion and physical harm, they also come at a cost (e.g., reductions in mobility that limit foraging and mating opportunities). Here, we suggest that the presence of social support, insofar as it signals a valuable resource for recovery, may therefore serve to temper the perceived challenge of physical exertion and the costs of potential exhaustion and injury. In conjunction with clearly perceived benefits of peak performance (e.g., winning an Olympic gold medal), this can lead to a lifting of the self-regulatory ‘brakes’ of pain and fatigue (relative to conditions of no support), ultimately enhancing performance.

Research on placebo effects has illuminated the neurobiological pathways through which environmental cues and socially derived beliefs affect physical performance. Studies have shown that placebo analgesia is mediated by endogenous opioid and endocannabinoid activity in top-down pain modulation systems (Wager et al. 2007; Benedetti et al. 2011). Given the overlap in neurobiological pathways involved in pain and fatigue modulation (Davis and Bailey 1997; Noakes et al. 2005; Pollak et al. 2014), and the importance of both as self-regulatory mechanisms that govern strenuous physical outputs (Noakes 2012), researchers have recently investigated whether placebo performance enhancement during exercise is based on neuropharmacological mechanisms similar to placebo analgesia in clinical contexts (Pollo et al. 2008, 2011). Studies have found that placebo performance enhancement can be *partially* blocked by opioid antagonists, suggesting involvement of the endogenous opioid system, but also other systems that underpin endogenous analgesia and self-regulation during physical exertion, such as the endocannabinoid system (Benedetti et al. 2007). Both endogenous opioids and endocannabinoids have been implicated in the formation, maintenance, and signalling of social bonds (Trezza et al. 2010; Machin and Dunbar 2011; Inagaki et al. 2016; Tarr et al. 2017), indicating one possible pathway through which the presence of social support modulates pain, fatigue and physical performance in strenuous exercise.

Responses to available social support are unlikely to be consistent across individuals, however. Effects of social support on responses to stressors may be ontogenetically tuned according to developmental environments of support or deprivation. Research suggests that variation in responsiveness to social support may be explained by individuals’ need for social assurance from others and whether they tend to perceive daily interactions to be supportive (Lee and Robbins 1995; Cohen et al. 2015). Other research on the moderators of social support effects has identified neuroticism as an important personality factor that affects how social support is received. Neurotic individuals are more likely to focus on the potential social costs of receiving social support, making them less likely to experience its positive effects (Park et al. 2013).

Although research has shown that social support can influence perceptions of physical challenges and alter activity in self-regulatory mechanisms involved in governing physical outputs, it remains unclear how these purported changes in self-regulation affect performance during everyday activities such as strenuous physical exercise. Although results consistently indicate a positive effect of social support on performance, previous experimental studies have either focused on coordination and skill-based tasks (e.g., golf putting; Rees and Freeman 2010), or cued social support indirectly (e.g., using synchrony as a social cohesion cue; Davis et al. 2015). This study is the first to test the effects of social support – as operationalised in the social psychological literature (Rees and Freeman 2010; Heinrichs et al. 2003) – among everyday exercisers during a strenuous physical activity.

We tested the hypothesis that social support increases outputs during strenuous physical exercise while reducing perceptions of exertion and physical discomfort. In a between-subjects experiment, participants’ social support was manipulated before a difficult physical exercise task. Our social support manipulation was similar to those used in other studies where social support was induced by having a close friend accompany participants while they completed a stressful task (e.g., Heinrichs et al. 2003). However, it differs in that companions waited in another room while their partners completed the experimental task. This was done to avoid potential confounds arising from observation effects. It also differs in that participants in both the social support (hereafter ‘companion’) and control (hereafter ‘solo’) conditions brought a companion with them to the study (instead of only in the companion condition); this was done to ensure that participants (hereafter ‘exercisers’) had similar experiences and expectations leading up to participation in the study and immediately prior to the manipulation. The exercise task consisted of four 30-s, maximum effort cycling bouts; physical performance outputs and self-reports of physical discomfort and exertion were measured across the four bouts. Although an indirect measure, self-reports of physical discomfort have been shown to correlate with neurobiological activity in pain- and fatigue-processing neural systems (e.g., Eisenberger et al. 2011; Pollak et al. 2014). Furthermore, direct measurements of activity in endogenous opioid and endocannabinoid systems are invasive – involving lumbar puncture (Dearman and Francis 1983; Cohen et al. 2010) – or expensive – involving blood tests or PET scans (Boecker et al. 2008; Raichlen et al. 2012). We will therefore use pain and fatigue ratings in the context of a behavioural experiment as an initial test of our hypothesis about the effects of social support on anaerobic outputs and to inform conjectures of possible psychological and neurobiological mechanisms underlying these effects. Finally, we also measured a set of individual attributes that have been shown to alter the effects of social support – the quality of the exerciser-companion relationship, exercisers’ social support in daily life, their need for social assurance, and their level of neuroticism – as these variables had the potential to moderate the effect of our manipulation on strenuous physical exercise.

## Methods

### Participants

Participants were recruited in Oxford, UK for a study putatively “investigating the links among personality, exercise, and cognition.” To participate, exercisers were required to be “at least recreationally fit,” aged 18–49, and to bring a companion to the study. Companions were to be “someone you feel you have a close connection with and that you can depend upon in times of need (such as a friend, romantic partner, or family member).” Exercisers and their companions were compensated £15 and £10, respectively. This study was approved by the School of Anthropology and Museum Ethnography Research Ethics Committee (Reference number: SAME/CUREC1A/15–045).

An initial power analysis – assuming a medium to large effect size (${\upeta}_{\mathrm{p}}^2$ = .13) with a power of 0.8 and an α equalling .05 – generated a recruitment goal of 80 pairs of participants. In total, 81 exercisers and their companions participated in the study. We excluded four pairs from our analyses: one due to experimenter error, one because of an underage companion, and two for correctly guessing the hypothesis and experimental condition. This left us with a total of 77 exercisers (53 females, age range = 18–42 years, *M* = 23.60 years, *SD* = 5.44 years), and their companions. Additionally, two exercisers (one in each condition) did not finish their fourth bouts of exercise due to feeling unwell. Thus, there were missing data for their exercise bouts. These two exercisers were excluded only from the mixed ANOVAs reported below, as these models cannot accommodate missing data points. Data collection did not continue after data analysis.

### Procedures and Materials

Regardless of their allocated experimental condition, exercisers came to the study with their companions. Upon their arrival, participant pairs identified themselves as either exercisers or companions, after which they were led to separate rooms to read and sign informed consent forms. Exercisers were also asked to fill out the Physical Activity Readiness Questionnaire (Thomas et al. 1992): all were cleared to exercise. Exercisers began wearing a Polar H7 heart rate sensor with Bluetooth data recording from this point onwards. Average heart rates were calculated for each of the 30-s exercise bouts.

Immediately following this, and still in separate rooms, both participants answered a series of background questions related to their demographics and relationship with one another. Only the answers of exercisers were included in analyses. Participants were asked to provide their age, sex, nationality, and English language proficiency (*Fluent*, *Advanced*, *Intermediate*, or *Beginner*). Participants were also asked to answer the following question on their relationship with each other using seven-point Likert scales (1 – *Low amount*; 4 – *Medium amount*; 7 – *High amount*): “To what extent do you feel your companion is there for you when you need them (for example, emotionally, psychologically and/or materially)?”, “Rate your overall similarity to your companion.”, “Rate how connected you feel to your companion.”, and “Rate how close your relationship is with your companion.” Additionally, we asked participants how long they had known each other (years, months). Participants also answered the question: “How frequently are you in contact with your companion each week (whether in person, text, phone call, email, etc.)? Please indicate which of the following options best approximates the average number of times you are in contact with your companion each week. (*Less than once a month*, *A few times a month*, *Every week*, *A few times a week*, *Every day*, or *A few times a day*). An abbreviated version of the ‘Big Five’ personality inventory was used to measure participants’ personality traits (Rammstedt and John 2007). In order to obtain estimates of exercisers’ pre-experiment anaerobic fitness levels, a modified version of the Habitual Physical Activity Questionnaire was administered (see 1.1 in Electronic Supplementary Material) (Baecke et al. 1982).

Social support was manipulated after the pre-exercise questions, creating two, between-subjects conditions – companion and solo. In the solo condition, exercisers (still in a separate room) were informed that their companion had left after finishing their portion of the study. Exercisers in the solo condition were then told they would complete the rest of the study on their own. Exercisers in both conditions were then given instructions for the cycling bouts. In the companion condition, as a means of increasing exercisers’ perceptions of emotional and instrumental support (Rees and Freeman 2010; Heinrichs et al. 2003), the companion was brought into the room to listen to the instructions to ‘better understand’ the task, and exercisers were told their companion would be waiting for them in the next room should they “need anything at all” while they completed the rest of the study. A hypothesis-blind experimenter delivered the manipulation and instructions throughout the exercise portion of the experiment (see 1.2 and 1.3 in Electronic Supplementary Material).

The exercise task was based on the Wingate Anaerobic Test, a common measure of anaerobic fitness (Zupan et al. 2009; Bar-Or 1987). It consisted of a four-minute warm-up and practice session followed by four 30-s, maximum-effort cycling bouts (hereafter ‘exercise bouts’) on a Monark 874E ergometer (McArdle et al. 2010). Before each of the four exercise bouts, exercisers were reminded that it was “crucial” they give maximum effort (see 1.3 in Electronic Supplementary Material). The main dependent variable of interest was total anaerobic outputs over the four 30-s exercise bouts (a measure of response to ‘supramaximal’ exercise used in previous research; e.g., Bar-Or 1987; Greer et al. 1998). Total anaerobic outputs were calculated using the equation (McArdle et al. 2010):

$$ \mathrm{AW}\ \left(\mathrm{J}\right)=6g\left(\mathrm{kp}\right)\mathrm{r}\kern0.5em \left(\mathrm{in}\ 30\ \mathrm{seconds}\right) $$

where AW is total anaerobic work (or output) in joules, 6 is the distance travelled per pedal revolution in metres, *g* is gravitational acceleration in metres per second squared, kp is the resistance applied to the wheel in kilograms (6.5% of the exerciser’s body mass), and r is the total number of complete pedal revolutions during the 30-s bout. Between each exercise bout there was a three-minute recovery period during which, as a means of estimating activity in pain and fatigue modulatory systems, exercisers completed measures related to how much effort they felt they were giving and their levels of physical discomfort. For estimates of physical effort, exercisers answered the following prompt using the Borg Scale of Perceived Exertion (Borg 1982): “Please rate your feelings of exertion (how much physical effort you felt you were giving) during your last cycling bout.” For estimates of physical discomfort, exercisers answered the following prompt using a 20-point scale adopted from previous research on experimentally induced pain (Eisenberger et al. 2011): “Please rate the physical discomfort you felt during your last cycling bout. Look at the scale and the expression and then choose a number.”

Following a final recovery period after the fourth exercise bout, exercisers answered manipulation-check questions and a hypothesis probe. Manipulation checks included the following questions, all answered on seven-point Likert scales: “How much did you like the experimenters?” (1 = *not at all*, 7 = *a lot*), “Do you think you could have tried harder during the cycling bouts?” (1 = *not at all*, 7 = *very much so*), “How much did you enjoy the experiment?” (1 = *not at all*, 7 = *a lot*), “Did you feel self-conscious during the exercise portion of the experiment?” (1 = *not at all*, 7 = *very much so*), “How comfortable and supported did you feel during the exercise portion of the experiment?” (1 = *not at all*, 7 = *a lot*), “How anxious did you feel both before and during the experiment?” (1 = *not at all*, 7 = *a lot*), “How did you come to the experiment (walking, biking, by car, by bus, etc.)?” (text box for open response); “Did you and your friend come to the study together?” (*yes* or *no*), and “How often do you exercise with the companion you brought to the experiment?” (1 = *never*, 7 = *a lot*). The hypothesis probe was: “What do you think this experiment was about?” (text box for open response). Exercisers then answered the Multidimensional Scale of Perceived Social Support (MSPSS; Zimet et al. 1988) and the Social Assurance Scale (SAS; Lee and Robbins 1995). The MSPSS and SAS should be generally unaffected by exercisers’ experiences during the experiment, as both measures are about respondents’ personality characteristics and perceptions of social support in their daily lives (for a full schedule of measurements see 1.4 in Electronic Supplementary Material).

#### Data Availability

All data are available from the figshare database (10.6084/m9.figshare.5675392).

## Analyses

Firstly, Mann-Whitney *U* tests (used because of non-normal distributions of condition-wise differences in the outcome variables) were conducted to check the effects of the social support manipulation and to identify potential confounds. A mixed ANOVA was then used to test the hypothesis that exercisers in the companion condition would produce greater total anaerobic outputs over the four 30-s exercise bouts. A secondary mixed ANOVA on peak power during each 30-s bout, another commonly used output variable for Wingate Anaerobic Tests (McArdle et al. 2010), was also conducted as a means of confirming that observed patterns of results were not simply an artifact of analysing total anaerobic outputs as the main outcome variable (methods for this analysis are reported in 1.5 of the Electronic Supplementary Material). Mixed ANOVAs were also used to test the effects of the experimental conditions, exercise bouts, and their interaction on exercisers’ rates of perceived exertion (RPE) and reported physical discomfort, as well as their average heart rates during the 30-s exercise bouts. Finally, individual differences known to alter the receipt of social support were included in a series of mixed ANOVAs in order to identify moderators of the effects of social support on strenuous physical exercise outputs and experiences. Moderator variable scores were tertile split; analyses excluded the middle tertile as previous research suggests that social support differentially influences those scoring relatively high or low on these variables (Park et al. 2013; Cohen et al. 2015). All mixed ANOVAs included exerciser sex and estimated anaerobic fitness as control variables.

## Results

Regarding the manipulation checks and potential confounds, Mann-Whitney *U* tests revealed no significant differences between the two experimental conditions in exercisers’ post-exercise ratings of enjoyment, liking of the experimenters, self-consciousness, comfort and support, anxiety, or whether they felt they could have tried harder during the exercise bouts. There were also no significant between-condition differences in the exerciser-companion relationship, estimates of exercisers’ self-reported anaerobic fitness, personality traits, age, or sex (see Table S1 in Electronic Supplementary Material).

**Fig. 1:**
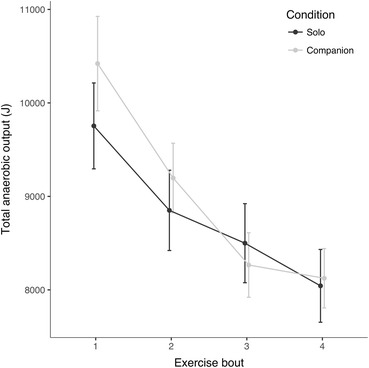
Mean (± 1 SE) total anaerobic output (joules, J) by condition

A mixed ANOVA was used to analyse exercisers’ total anaerobic outputs by experimental condition and exercise bout. The model, which included exerciser sex and estimated anaerobic fitness as control variables, revealed non-significant main effects of experimental condition and exercise bout, but a significant condition × exercise bout interaction, *F*(2.094, 213.0) = 5.344, *p* = .005, ${\upeta}_{\mathrm{p}}^2$ = .070 (see Fig. 1 and Table S2.1 in Electronic Supplementary Material). Post-hoc contrasts revealed that, compared to the solo condition, the presence of companion support led to higher initial total anaerobic outputs and steeper declines over the four exercise bouts (see Table S2.2 in Electronic Supplementary Material for post-hoc contrast summaries and effect sizes). Analysing peak power outputs instead of total anaerobic outputs yielded a similar pattern of results (see Table S3.1 and S3.2 in Electronic Supplementary Material). Another mixed ANOVA, including the same controls as above, revealed no effect of condition, exercise bout, or their interaction on average heart rates during the 30-s exercise bouts (see Table S4 in Electronic Supplementary Material).

**Fig. 2:**
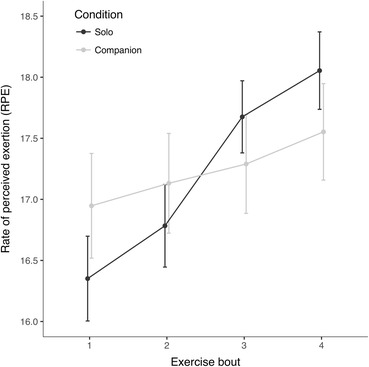
Mean (± 1 SE) rate of perceived exertion (RPE) by condition

Mixed ANOVAs were also used to analyse exercisers’ RPE and reported physical discomfort. This revealed a non-significant main effect of experimental condition, but a significant main effect of exercise bout, and a condition × exercise bout interaction on exercisers’ RPE, *F*(1.912, 139.583) = 5.343, *p* = .007, ${\upeta}_{\mathrm{p}}^2$ = .068 (see Fig. 2 and Table S5.1 in Electronic Supplementary Material); post-hoc contrasts showed that, compared to those in the companion condition, exercisers in the solo condition displayed greater increases in RPE across the four exercise bouts (see Table S5.2 in Electronic Supplementary Material). Regarding exercisers’ physical discomfort, analyses revealed a main effect of exercise bout only, *F*(1.784,130.241) = 31.854, *p* > .001, ${\upeta}_{\mathrm{p}}^2$ = .304; physical discomfort decreased across bouts (Fig. S1 and Table S6 in Electronic Supplementary Material).

**Fig. 3:**
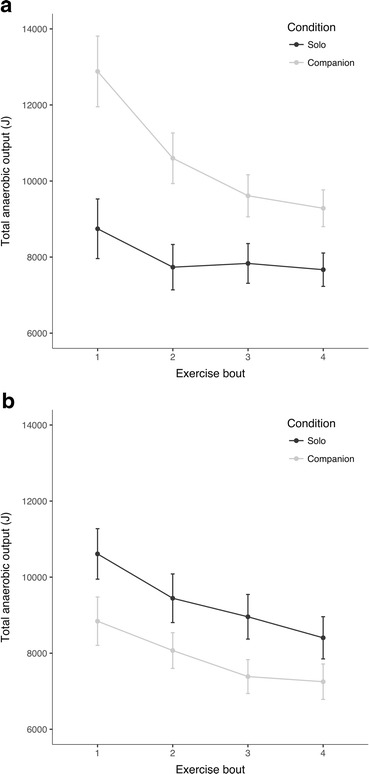
Mean (± 1 SE) total anaerobic output (joules*,* J) by condition for exercisers (**a**) low and (**b**) high in neuroticism

To further explore the condition × exercise bout interaction, four additional mixed ANOVAs were conducted. In addition to the control variables of exerciser sex and estimated anaerobic fitness, one of four potential moderators were also included: a component extracted from the questions on the exerciser-companion relationship (see 2.1 in Electronic Supplementary Material for information on the creation of this component), exercisers’ support in daily life, their need for social assurance, and their level of neuroticism. Bonferroni-adjusted significance levels of α = .0125 (.05 ÷ 4) were used as a means of correcting for multiple testing. Results revealed non-significant condition × exercise bout × moderator interactions for all models (Table S7 – Table S9 in Electronic Supplementary Material) except that including neuroticism, *F*(1.983,126) = 5.693, *p* = .005, ${\upeta}_{\mathrm{p}}^2$ = .119 (see Table S10.1 in Electronic Supplementary Material). Compared to the solo condition, the companion condition yielded higher total anaerobic outputs amongst exercisers low in neuroticism and lower total anaerobic output amongst exercisers high in neuroticism (see Fig. 3 and Table S10.2 in Electronic Supplementary Material).

The assumption checks for the models reported above can be found in the Electronic Supplementary Material (Section 3).

## Discussion

This experiment investigated the influence of social support on performance in a difficult exercise challenge. Due to non-significant between-condition effects on mean performance overall (i.e., all exercise bouts collapsed into a single mean), the null hypothesis of no overall difference in performance between the two conditions cannot be rejected. However, analyses revealed a condition × exercise bout interaction on exerciser outputs, with higher initial outputs and steeper declines over the four exercise bouts in the companion condition, compared to the solo condition.

Higher initial outputs and steeper declines over time in the companion condition, compared to the solo condition, are congruent with previous findings from disparate fields of research. Within social psychology, experiments have shown that perceptions of physical and social resources and physiological capabilities can affect how challenges are assessed, altering participants’ perceptions of their ability to succeed. For example, research has shown that variables that can impede physical performance – such as additional weight to carry, fatigue, or poor health – can cause distances to be perceived as greater (Bhalla and Proffitt 1999). In relation to the present study, social support has been shown to increase perceptions of self-efficacy amongst exercisers (Christensen et al. 2006), which may help to explain the finding that physical challenges are perceived as less difficult in the presence of a supportive companion (Schnall et al. 2008). In the context of this experiment, supported exercisers may have estimated the exercise task to be easier, which could have led to greater initial outputs, followed by lower outputs in later exercise bouts because of greater accrued muscle fatigue.

Nevertheless, it is important to consider why a higher level of output was not sustained, given that the rationale for our hypothesis was that the presence of social support would alter activity in the neurobiological mechanisms governing pain, fatigue, and physical outputs, all of which are already known to be susceptible to environmental cues, such as ergogenic placebo treatments (e.g., Benedetti et al. 2007; Pollo et al. 2008, 2011). It is unknown whether ergogenic placebo studies would produce similar output patterns via activity levels in endogenous pain and fatigue modulation systems – to date, no studies of comparable design have been conducted. One possible explanation, albeit speculative, concerns the interaction between the central and peripheral neurobiological mechanisms that govern outputs during strenuous physical exertion. Research using opioid analgesics has shown patterns of decline similar to the total anaerobic outputs of exercisers in this study. Lumbar intrathecal injections of the opioid agonist fentanyl before a maximum-effort 5 km cycling trial led to increased outputs during the first 2.5 km, and decreased outputs in the second 2.5 km, compared to placebo injections (Amann et al. 2009). These results were hypothesised to result from an opioid mediated ‘block’ on fatigue-signalling locomotor muscle afferents in the fentanyl condition. This block would reduce the inhibitory influence of these muscle afferents – and the pain and fatigue they signal – on central motor drive (Pollak et al. 2014), causing increased initial outputs but then also greater locomotor fatigue. Ultimately, relatively higher accumulations of muscle fatigue from increased first-half outputs in the fentanyl condition led to reduced muscle drive and outputs in the second half of the trials, relative to the placebo condition.

This result allows for speculation on how the presence of close, supportive others could similarly affect exercise outputs. The output profiles in the companion condition of the present study may result from processes involving heightened activation in endogenous, self-regulatory systems that reduce perceptions of pain and fatigue and that are also known to be involved in signalling the presence of social bonds (Inagaki et al. 2016; Fattore et al. 2010), such as the endogenous opioid and endocannabinoid systems (Panksepp et al. 1981; Wager et al. 2007; Benedetti et al. 2011). As a result of reduced pain and fatigue signalling, exercisers in the companion condition – who potentially also viewed the task as less challenging (Schnall et al. 2008) – may have ‘overshot’ optimum outputs initially, producing higher outputs in the first two bouts, but, due to heightened locomotor fatigue, lower outputs in the final two bouts. Future research should explore the interplay between central motor drive, afferent inputs from locomotor muscles, and endogenous pain and fatigue modulating systems using manipulations involving both ergogenic placebos and social support (Tanaka and Watanabe 2012).

Performance outputs can also be interpreted alongside consideration of other direct physiological and self-report measures. Between-condition differences in exercisers’ output profiles were not reflected in heart rate data, which showed no significant effects of experimental condition or exercise bout. Furthermore, in the solo condition, RPE increased across bouts in parallel with decreased outputs, while in the companion condition, RPE remained relatively constant. This could be explained in a variety of ways, including reduced metabolic costs and perceived effort in socially supportive conditions. In the companion condition, these effects may have been underpinned by lower levels of negative affect (Lane et al. 2011) and reduced locomotor muscle feedback owing to an endogenous opioid-based blockade of signals from muscle afferents; exercisers in this condition may have thus experienced reduced sensitivity to variation in RPE (Amann et al. 2009). Ultimately, however, differences in RPE profiles between the two conditions were small, with responses varying by only one to two points on the 14-point RPE scale (see Fig. 2).

Other factors may account for the observed results. For example, relatively steeper output declines in the companion condition are also potentially attributable to a decrease over time in the perceptual salience of social support. Reported social support was measured only once, after completing the full exercise task, so this remains speculative. Regardless, exercisers’ perceptions of social support could potentially be improved by adding reminders of companion support during or between the exercise bouts. Relatedly, self-reported perceived social support did not differ between conditions. However, the effects of support on self-regulation are not necessarily contingent on explicit awareness and appraisal of support, but rather by implicit cognitive processes (e.g., Kikusui et al. 2006; Cohen et al. 2015). Future studies could incorporate additional cognitive or behavioural measures that have robust associations with the presence or absence of perceived social support.

The effects of social support on physical outputs are likely strongest when individuals are performing closest to their physiological maximum, such as when the potential benefits are high. Adaptive self-regulation of physical output in exercise is calibrated by perceived costs and benefits, and the resources available to maximise the latter while minimising the former. Our study design instructed all exercisers to engage in a maximum effort activity, following relevant precedents in the sports performance literature. However, given that our sample consisted of untrained athletes, it is possible that exercisers were not accustomed to or capable of maximal effort, as learning how to overcome the neurological restraint mechanisms involved in strenuous physical exercise can take years of training. Therefore, exercisers may not have performed at a level of effort at which the buffering effects of social support would potentially be at their strongest. Future studies could improve on this by using a subset of the population that is accustomed to giving maximal effort under laboratory conditions, such as trained athletes.

A final possibility suggested by the results of this study is that differences in personality may systematically influence the support-performance relationship. This is in accordance with previous research, which identified the personality factor neuroticism as an important moderator of the effects of social support (Park et al. 2013). Here, neuroticism was found to moderate the effects of social support on anaerobic performance. Compared to their counterparts in the solo condition, exercisers in the companion condition that were low in neuroticism produced greater total anaerobic outputs across all exercise bouts. In fact, these exercisers averaged 10,594 joules per bout, compared to an average of 8604 joules per bout for all other exercisers (see Fig. 1). Exercisers high in neuroticism showed the opposite pattern; compared to their counterparts in the solo condition, neurotic exercisers in the companion condition produced lower total anaerobic outputs across all exercise bouts. Although the sample size following the tertile split was relatively small (*n* = 48) – and results should therefore be treated with caution – these differences were highly significant and the effect of the interaction was strong (${\upeta}_{\mathrm{p}}^2$ = .119). This pattern of results is in accordance with previous research, which found that the positive effects of social support vary according to neuroticism levels, with those low in neuroticism being the most susceptible to beneficial social support effects (Cohen et al. 2015; Park et al. 2013). Due to their preoccupations with the potential costs of receiving social support (e.g., being in indebted to or inconveniencing supporters), highly neurotic exercisers may have been less susceptible to the buffering effects of social support on the pain, fatigue, and negative affect surrounding physical challenges (Park et al. 2013; Cohen et al. 2015; Beedie et al. 2012).

Research has shown that neuroticism correlates with early life stress and fast life history strategies (Figueredo et al. 2005); relatively reduced social buffering among neurotic individuals can potentially be explained by behavioural strategies that are adaptively tuned during development to environments where the benefits of social support are less likely to be received. The results of the present study and others on the relationship between social support and personality therefore tentatively suggest a reasonable modification of our hypothesis, such that among individuals who are sensitive to the benefits of social support, companion presence has positive effects on physical performance. This interpretation has parallels in clinical placebo research, where individual differences in placebo responsiveness are well established and neuroticism has been shown to negatively predict placebo analgesia (Peciña et al. 2013).

### Conclusions

This experimental study is the first to examine causal links between social support and performance among ordinary exercisers, using a novel experimental method that manipulates the presence of social support without introducing confounding audience effects. Results are situated within an interdisciplinary account of the trade-offs involved in ultimate functions of optimal self-regulation during physical exertion and the proximate, neurobiological mechanisms that underpin this self-regulation. The findings suggest that social support influenced exercisers’ self-regulatory responses to strenuous physical exertion, leading to greater initial outputs and steeper declines over time. The results also suggest that the positive effects of social support were strongest among exercisers low in neuroticism.

Although the results of this experiment are promising, they should be replicated in future studies with larger, more diverse samples, while testing the specific mechanisms hypothesised to underlie these observed effects. For example, given the importance of pacing strategies in completing four consecutive 30-s maximum effort exercise bouts, a replication with more experienced athletes might result in different pacing strategies and output profiles. Additionally, pharmacological blocking methods have been used to test the effects of synchrony on the endogenous opioid system (Tarr et al. 2017). These methods could be used to test the involvement of the endogenous opioid system in social support effects on the self-regulation of physical outputs during anaerobic exercise, and whether or not heightened endogenous opioid activity explains greater initial outputs and steeper declines over time under conditions of social support. Future work could also take into account other direct measures of performance in addition to power outputs and heart rate. Additional direct measures include blood glucose and oxygen uptake, both of which have been shown to vary in response to environmental cues and their emotional responses (Beedie et al. 2012). This could reveal whether supported participants produce similar overall outputs to controls, but at lower metabolic costs, which would be in accordance with the condition × exercise bout interaction obtained for exerciser RPE. Regarding the lack of hypothesised differences in reports of social support between conditions, more research into the effects of implicit expectations of social support could also augment existing clinical literature on placebo effects via explicit knowledge versus expectation (e.g., about treatment efficacy from medical institutions; Benedetti 2009; Kaptchuk 2011). As relevant implicit expectations and biases often result from long-term developmental and social experiences that may vary across individuals and cultures, this work could also inspire future research into the life history and personality-dependent effects of perceived social support on pain, fatigue, physical performance, and self-regulation more generally.

The hypothesis-driven approach developed here offers new methods for testing the effects of social support and motivates new questions and directions for research that will potentially shed light on causes, incidence, and limits of social support effects occurring not just in health, but in everyday physical activity.

## Electronic supplementary material

Electronic Supplementary Material (ESM) 1 (PDF 885 kb)
