# Supplementary material for: The Effects of Social Support on Strenuous Physical Exercise
Source: Adapt Human Behav Physiol. 2018 Jan 11;4(2):171–87. doi: 10.1007/s40750-017-0086-8 (PMC5935032; doi:10.1007/s40750-017-0086-8)
Supplement: Supplementary file 1 — (PDF 885 kb) [file 40750_2017_86_MOESM1_ESM.pdf]

Electronic Supplementary Material

The Effects of Social Support on Strenuous Physical Exercise

Adaptive Human Behavior and Physiology

Arran Davis[1]* and Emma Cohen[1,2]

[1] Institute of Cognitive and Evolutionary Anthropology, University of Oxford, 64 Banbury Road, Oxford, OX2 6PN, UK [2] Wadham College, Parks Road, Oxford, OX1 3PN, UK

*davis.arran@gmail.com / arran.davis@anthro.ox.ac.uk | +44 (0)7721 513026 | ORCID: 0000-0002-8561-7768

1. Procedures and measurements

1.1. Anaerobic fitness estimate

To estimate exercisers' anaerobic fitness we used a slightly extended version of the sport index in the Habitual Physical Activity Questionnaire (Baecke, Burema, & Frijters, 1982). Our alteration was to allow exercisers to list four sports instead of two in order to better estimate time spent doing anaerobic activities. Thus, we used the following formula:

$$\text{Anaerobic fitness} = \sum_{i=1}^{4} (\text{intensity} \times \text{time} \times \text{proportion})$$

Where, according to Baecke et al. (1982):

Intensity is 0.76 for low intensity sports, 1.26 for medium intensity sports, and 1.76 for high intensity sports.

Time is 0.5 for less than 1 hour per week spent playing the sport, 1.5 for 1-2 hours per week, 2.5 for 2-3 hours per week, 3.5 for 3-4 hours per week, and 4.5 for greater than 4 hours per week spent playing the sport.

Proportion is 0.04 for less than 1 month per year playing the sport, 0.17 for 1-3 months per year, 0.42 for 4-6 months per year, 0.67 for 7-9 months per year, and 0.92 for greater than 9 months per year playing the sport.

A score of 0 was given when no sport was listed.

1.2. Social support manipulation

The following table summarises the social support manipulation, given verbally by a hypothesis blind experimenter:

**Solo condition**	**Companion condition**
"Okay, good. So, you've finished the questionnaire part of the study. Your companion also finished their questionnaire and has left. You will do the strenuous exercise part of the study on your own. I'll now give you the instructions for the strenuous exercise you will be doing. After I give you these instructions I will give you a brief recap. After this you will begin the strenuous exercise portion of the study."	"Okay, good. So, you've finished the questionnaire part of the study. Your companion also finished their questionnaire. I'll now give you the instructions for the strenuous exercise you will be doing. While I do this your companion will be reading a version of the instructions you will be receiving - even though they will not be doing the strenuous exercise part of the study, they will understand what it entails. After I give you these instructions I will give you a brief recap. Your companion will come into the exercise room to observe this recap of your instructions so that he/she can better understand what you will be doing. After this you will begin the strenuous exercise portion of the study."
Pre-exercise instructions are given to the exerciser (see 1.3.2).	
Exerciser is given a recap (see 1.3.3) of the instructions.	The companion is brought into the room with the exerciser and they are both given a recap (see 1.3.3) of the instructions.
"Okay, you will now begin the strenuous exercise portion of the study."	"Okay, your companion is going to go back to their seat in the next room. They will be there for you if you need anything at all and will wait for you there until you are finished. You will now begin the strenuous exercise portion of the study."
While participating in the exercise portion of the study the exerciser is given instructions on how to complete the warm-up and maximum-effort cycling bouts (see 1.3 below).	

1.3. Exercise trial procedure and measures

1.3.1. Procedure

The cycling bouts involved pedalling for a set amount of time at a fixed amount of resistance, 6.5% of the exerciser's body weight (McArdle, Katch, & Katch, 2010), on a Monark 874E ergometer (hereafter 'stationary cycle').

Exercisers began the exercise portion of the study with a four-minute warm-up on the stationary cycle. During the warm-up exercisers cycled at their chosen pace. At two and three minutes into the warm-up they practiced the 10-second flying start (see below) followed by 10 seconds of maximum effort cycling at their respective resistance.

Following the warm-up exercisers were given a three-minute break before beginning their first of four 30-second cycling bouts. Before beginning each bout, exercisers were reminded that it was "crucial" they give maximum effort during the 30-second bouts.

The test began with a 'flying start' in which exercisers cycled as fast as possible at no resistance for 10 seconds before their body-weight adjusted resistance was applied. The experimenter counted down aloud the final five seconds of the flying start ("5-4-3-2-1-GO") and applied the resistance while saying "GO". The trial began at "GO" and ended with a 5 second countdown ("5-4-3-2-1-STOP").

After each bout exercisers were given a three-minute break during which they completed the Borg Scale of Perceived Exertion (Borg, 1982) and indicated the amount of physical discomfort they felt during the exercise bout they had just completed using a 20-point scale adopted from previous research on experimentally induced pain (Eisenberger et al., 2011). Exercisers answered these questions at a standing desk next to the stationary cycle.

The experimenter notified exercisers when their three-minute break was almost finished and asked them to return to the stationary cycle to begin their next maximum-effort bout.

Exercisers repeated this procedure until they had completed four maximum-effort, 30-second cycling bouts in total.

1.3.2. Pre-exercise instructions

These instructions were read to exercisers before the social support manipulation took place:

"This is the cycle on which you will complete the maximum effort exercise bouts. This basket holds the weights that work to apply resistance. When I drop the basket

resistance is applied. The amount of weight to be applied depends on how much you weigh.

“Each maximum effort cycling bout will last 30 seconds. Before the 30-second period begins, you will have a 10 second period during which you will pedal to move this wheel as fast as possible. I will count you into the beginning of the 30-second period with ‘5-4-3-2-1-GO’. When I say ‘go’ I will drop this basket, and this will apply resistance to the wheel. This is the beginning of the 30-second bout. You will pedal at maximum effort for the next 30 seconds. You will feel fatigue and discomfort in your legs, but should try your hardest to keep pedalling as fast as you can. I will count down the final five seconds of the 30-second period with ‘5-4-3-2-1-STOP’. When I say ‘stop’ you are to stop pedalling.

“You will then be given a three-minute break during which you will answer some questions on the computer. After you finish this you will start the next cycling bout as described above.

“Before you begin you will do a four-minute warm-up at no resistance. Two times during this warm-up [at the two and three-minute mark] I will drop the basket and you will do 10 seconds of maximum effort cycling to get used to exercising at the level we want. After the warm-up you will answer questions on the computer as well. Then you will do the maximum-effort cycling bouts.”

1.3.3. Pre-exercise recap

This recap was read to exercisers either in the presence (companion condition) or absence (solo condition) of their companion:

“I’ll now give you a quick recap of what you will be doing. The exercise portion of the study consists of 30-second maximum effort cycling bouts on this stationary cycle. As I said, this is the basket that will give you a resistance that depends on your weight. When I pull the basket up there is no resistance. Dropping the basket adds resistance. Before each 30-second cycling bout you will have a 10-second warm-up in which you are to start pedalling as fast as possible. Five seconds in to this warm-up – so 5 seconds before you begin the 30-seconds of maximum effort – I will count down from 5 and when I say ‘go’ I will drop the weight basket and your 30-second cycling bout will begin. I will count down the final five seconds of the 30-second period with ‘5-4-3-2-1-STOP’. When I say ‘stop’ you are to stop pedalling. Remember, you have to give maximum effort despite feelings of fatigue and discomfort in your legs. After the 30 seconds of cycling you will have a three-minute recovery period in which you will remain standing while you answer some questions at your computer. I’ll let you know when your three minutes is up and you will do another 30-second maximum-effort bout with a 10-second warm-up, the same as last time. Then, you’ll again have three minutes to rest and answer questions. We’ll repeat this procedure until you are done with the exercise portion of the study.”

1.3.4. Instruction for exercise warm-up and trials

These instructions were read to exercisers immediately before and during the exercise trials:

“Okay, you can now get on the cycle and begin warming up at a pace that is comfortable to you. At two minutes and three minutes I will drop the weight basket and you will practice giving maximum effort.

“Okay, you may begin.”

At two minutes: “Okay, I am going to drop the weight basket now, when I say go pedal as hard as you can. 5-4-3-2-1-GO!”

After 10 seconds: “Good job, but I think you could pedal a bit harder. Continue pedaling until the three-minute mark and I will drop the weight basket again.”

At three minutes: “Okay, I am going to drop the weight basket again, when I say go pedal as hard as you can. 5-4-3-2-1-GO!”

After 10 seconds: “Okay, that is better but during the 30-second trials I need you to try even harder – as hard as you possibly can.”

At four minutes: “Okay that is the end of the warm-up, please go to the computer and answer the questions on the screen. You will begin the 30-second maximum effort cycling bouts after a three-minute rest period.”

After three minutes: “Okay, you will now begin your first 30-second bout of maximum effort cycling. Remember there is a 10 second ‘warm-up’ period of cycling before the 30-second maximum effort bout, and I'll count you down from five seconds. When I say ‘go’ I will drop the weight basket and your 30 seconds will begin. Remember, it is crucial that you give maximum effort during these 30 seconds. This is the first of four 30-second bouts you will be doing today. Are you ready you ready to begin? Okay, begin.”

After five seconds: “5-4-3-2-1-GO!”

After 25 seconds: “5-4-3-2-1-STOP!”

After the first trial: “Okay good, you’ve completed one of the four 30-second exercise bouts. Please go to the computer and follow the directions on the screen. Remember to catch your breath and rest; you have three minutes until the next trial.”

After three minutes: “Okay, your three-minute rest period is up. Please get back on the bike. You will now begin your second 30-second bout of maximum effort cycling. The procedure is the same as last time. Are you ready to begin? Okay, being.”

(This continues for the next two trials – same instructions)

After the fourth trial: “Okay, you have finished your last 30-second bout of exercise. Please go to the computer and follow the directions on the screen. Remember to catch your breath and rest.”

1.4. Schedule of measurements

Variable	Question type
Pre-exercise questions (both exerciser and companion)	
Demographic information	Age, sex, nationality, and English proficiency.
Opinions of exerciser-companion relationship	Similarity, closeness, connectedness, and how much the other is there for them in times of need (seven-point Likert Scales).
Duration of exerciser-companion relationship and frequency of contact	Length of relationship (years, months); frequency of contact (multiple choice).
Personality	10-item Big Five measure (Rammstedt & John, 2007).
Anaerobic fitness	Modified version of Habitual Physical Activity Questionnaire (see 1.1).
Exercise affect questions – presented after each cycling bout (exerciser only)	
Rate of perceived exertion (RPE)	Borg Scale of Perceived Exertion (Borg, 1982).
Physical discomfort	Amount of physical discomfort on a 20-point scale (Eisenberger et al., 2011).
Post-exercise questions (exerciser only)	
Manipulation checks	Feelings of enjoyment, comfort and support, and anxiety during the study, liking of the experimenters, and degree to which they could have tried harder (seven-point Likert Scales).
Hypothesis probe	Guess experimental hypothesis (text box for open response).
Perceptions of daily social support	Multidimensional Scale of Perceived Social Support (Zimet, Dahlem, Zimet, & Farley, 1988).
Need for social support and assurance	Social Assurance Scale (Lee & Robbins, 1995).

1.5. Peak power calculation

Peak power outputs were calculated using the equation (McArdle et al., 2010):

$$PP\ (W) = 6g(kp)r / 5$$

where PP is peak power output in watts, 6 is the distance travelled per pedal revolution in metres, *g* is gravitational acceleration in metres per second squared, kp is the resistance applied to the wheel in kilograms (6.5% of the exerciser's body mass), and r is the total number of complete pedal revolutions during the five seconds of the 30-second exercise bout where the exerciser produced the greatest number of pedal revolutions (this is usually, but not necessarily, the first five seconds of the bout).

2. Analyses

2.1. Exerciser-companion relationship component

Examination of scree plots, communalities after extraction, and internal consistency tests from a principal components analysis suggested that one component (comprised of the questions related to closeness, connectedness, and how much exercisers felt their companion was there for them in times of need) should be extracted from the four questions measuring exercisers' opinions about their relationship with their companion (see 1.4). The question on exercisers' similarity to their companion had low correlations with the questions in the component, and was thus dropped from further use. A subsequent analysis revealed that the component explained 82.26% of the variance in the three questions. Cronbach's α, based on standardised question scores, indicated good internal consistency for the exerciser-companion relationship component (α = .883).

3. Model assumptions

Here we report analyses meant to check the assumptions of the models reported in the main text. For the mixed ANOVAs, we tested the assumptions of normality (that that distribution of total anaerobic outputs was normally distributed in each cell), homogeneity of variance, sphericity, and homogeneity of inter-correlations (Field, Miles, & Field, 2012).

We only report those models where claims of statistical significance are made.

3.1. Main analysis of total anaerobic outputs

Regarding the normality of total anaerobic outputs within cells, all cells had approximately normal distributions, Shapiro-Wilk normality tests (all *W*'s > 0.956 and *p*'s > .05), except for bouts 2 ($W = 0.925$ and $p = .015$), 3 ($W = 0.905$ and $p = .004$), and 4 ($W = 0.929$ and $p = .021$) of the solo condition, although examination of normal Q-Q plots (see Figures S2.1 – 2.3) revealed that these deviations are rather small. Further, research has shown that the *F*-statistic is robust to non-normality when group sizes are roughly equal, as is the case with this analysis; there were 37 exercisers in the solo condition and 38 in the companion condition (Field et al., 2012; Glass, Peckham, & Sanders, 1972)

The homogeneity of variance assumption was met; Levene's tests revealed that variances in total anaerobic outputs between bouts over conditions were approximately equal (all $F(1, 73) < 0.400$, $p > .05$). A Box's *M* test revealed that the homogeneity of interclass correlations assumption was broken, *M*, $F(10, 25{,}434.126) = 33.508$, $p > .001$. However, this may have more do with the non-normality of total anaerobic outputs within cells (Morrison, 1998).Using a log transformation of total anaerobic outputs yields a model with more normally distributed data in each cell, similar results to the main mixed ANOVA reported in the main text (i.e., a significant exercise bout × condition interaction), and a Box's *M*, $F(10, 25{,}434.126) = 29.026$, $p = .002$, that previous research suggests to ignore: Morrison (1998) writes that, due to its sensitivity to deviations from normality, all Box's *M* tests with significance levels greater than $p = .0001$ should be ignored.

The sphericity assumption was violated for the repeated measures effect of exercise bout ($M = .536$, χ^2 $(5) = 43.421$, $p < .001$). Considering the Greenhouse-Geisser estimate of sphericity (.698), all *F*-ratios reported in the main text use the Greenhouse-Geisser correction (Field et al., 2012).

3.2. Analysis of physical discomfort

Regarding the normality of physical discomfort self-reports within cells, all cells had approximately normal distributions, Shapiro-Wilk normality tests (all *W*'s > 0.965 and *p*'s > .05), except bout 4 in the solo condition ($W = 0.939$ and $p = .043$) and bouts 3 ($W = 0.932$ and $p = .024$) and 4 ($W = 0.934$ and $p = .026$) in the companion condition, although examination of normal Q-Q plots (see Figures S3.1 – 3.3) revealed that these deviations are rather small. Further, research has shown that the *F*-statistic is robust to

non-normality when group sizes are roughly equal, as is the case with this analysis; there were 37 exercisers in the solo condition and 38 in the companion condition (Field et al., 2012; Glass et al., 1972).

The homogeneity of variance assumption was met; Levene's tests revealed that variances in total anaerobic outputs between bouts over conditions were approximately equal (all $F(1, 75) < 1.200$, $p > .05$). A Box's *M* test revealed that the homogeneity of interclass correlations assumption was broken, *M*, $F(10, 26{,}849.262) = 24.134$, $p = .012$, although this test is extremely sensitive to departures from multivariate normality, and researchers have suggested that tests with significance levels greater than $p = .0001$ be ignored (Box, 1949; Morrison, 1998).

The sphericity assumption was violated for the repeated measures effect of exercise bout ($M = .399$, χ^2 (5) = 67.676, $p < .001$). Considering the Greenhouse-Geisser estimate of sphericity (.645), all *F*-ratios reported in the main text use the Greenhouse-Geisser correction (Field et al., 2012).

3.3. Analysis of rates of perceived exertion (RPE)

Shaprio-Wilks tests revealed that the normality assumption had been broken (all *W*'s < 0.940 and *p*'s < .05); RPE was not normally distributed within cells (see Figure S4.1 – 4.8). However, research has shown that the *F*-statistic is robust to violations of the normality assumption when group sizes are roughly equal, as is here the case; 37 exercisers were in the solo condition and 38 were in the companion condition (Field et al., 2012; Glass et al., 1972).

The homogeneity of variance assumption was also broken, although, again, research has shown that ANOVA tests are robust to departures from this violation, especially when these departures are small and group sizes are roughly equal (Field et al., 2012; Glass et al., 1972). This is the case with this data (see above for group sizes), as evidenced by Levene's tests, which were only significant for exercise bouts 2, $F(1, 75) = 4.674$, $p = .034$, and 3, $F(1, 75) = 6.727$, $p = .011$, and plots of RPE variance between the solo and companion conditions across the four exercise bouts (see Figure S5).

A Box's *M* test revealed that the homogeneity of interclass correlations assumption was also broken, *M*, $F(10, 26{,}849.262) = 22.252$, $p = .021$, although this test is extremely sensitive to departures from multivariate normality, and researchers have suggested that tests with significance levels greater than $p = .0001$ be ignored (Box, 1949; Morrison, 1998).

Finally, the assumption of sphericity was also broken for the repeated measures effect of exercise bout ($M = .425$, χ^2 (5) = 63.126, $p < .001$). Considering the Greenhouse-Geisser estimate of sphericity (.666), all *F*-ratios reported in the main text use the Greenhouse-Geisser correction (Field et al., 2012).

Given that this model was particularly problematic – the assumptions about normality, homogeneity of variance, interclass correlations, and sphericity were all not met – we ran a robust mixed ANOVA using trimmed means on the same data (Wilcox, 2012). This provided similar findings to the mixed ANOVA reported in the main text; a main effect of exercise bout (Q = 13.149, p <.001), and a condition × exercise bout interaction (Q = 3.894, p = .016) on exercisers' RPE (see Table S11).

3.4. Moderation of total anaerobic outputs by exerciser neuroticism

Regarding the normality of total anaerobic outputs within cells, all 16 cells had approximately normal distributions, as evidenced by Shapiro-Wilk normality tests (all W's > 0.855 and p's > .05).

The homogeneity of variance assumption was met; Levene's tests revealed that variances in total anaerobic outputs between bouts over conditions were approximately equal (all F(3, 44) < 0.550, p > .05). A Box's M test revealed that the homogeneity of interclass correlations assumption was met, M, F(30, 3,972.035) = 30.313, p = .729.

The sphericity assumption was violated for the repeated measures effect of exercise bout (M = .371, χ^2 (5) = 40.405, p < .001). Considering the Greenhouse-Geisser estimate of sphericity (.661), all F-ratios reported in the main text use the Greenhouse-Geisser correction (Field et al., 2012).

Tables

Table S1 Manipulation Checks

**Variable**	**Scale**	**Solo condition *M* (*SD*)**	**Companion condition *M* (*SD*)**	**Mann-Whitney *U***	***p***
Age	Years	23.55 (5.99)	23.64 (4.94)	637.50	.288
Anaerobic fitness estimate	HPAQ	3.79 (3.23)	5.06 (3.80)	613.50	.193
Enjoyment	Seven-point Likert	4.32 (1.31)	3.72 (1.88)	584.00	.101
Liking of experimenters	Seven-point Likert	5.11 (1.34)	5.13 (1.74)	697.00	.647
Self-consciousness	Seven-point Likert	4.13 (2.16)	3.72 (2.03)	644.00	.317
Comfort and support	Seven-point Likert	5.21 (1.28)	4.74 (1.82)	661.00	.405
Anxiety	Seven-point Likert	3.18 (1.56)	3.41 (1.59)	691.00	.604
Tried harder during exercise bouts?	Seven-point Likert	3.11 (1.93)	3.08 (1.72)	731.50	.921
Perceived daily social support	MSPSS (Zimet et al., 1988)	66.34 (10.62)	70.41 (11.44)	569.00	.079
Need for social assurance	SAS (Lee & Robbins, 1995)	29.45 (9.80)	31.59 (9.93)	646.50	.335
Extraversion	Big 5 (Rammstedt & John, 2007)	6.19 (2.14)	7.13 (1.94)	302.50	.875
Agreeableness	Big 5 (Rammstedt & John, 2007)	7.32 (1.47)	6.36 (1.71)	223.00	.083
Conscientiousness	Big 5 (Rammstedt & John, 2007)	6.97 (1.60)	7.49 (1.60)	232.00	.120
Neuroticism	Big 5 (Rammstedt & John, 2007)	5.66 (1.91)	6.08 (1.89)	280.50	.551
Openness	Big 5 (Rammstedt & John, 2007)	7.66 (1.70)	7.64 (2.17)	296.50	.781
Length of relationship	Months	33.61 (48.86)	32.10 (38.34)	715.50	.795
Frequency of contact	Six-option multiple choice	5.05 (0.96)	5.15 (1.11)	664.00	.401
Relationship with companion	Component	-0.08 (1.13)	0.08 (0.87)	699.50	.672
Sex	The solo condition consisted of 12 males and 26 females. The companion condition consisted of 12 males and 27 females (χ^2 = .006, *p* = .939).				

Table S2.1 Results of main mixed ANOVA on total anaerobic outputs (see also Table S2.2)

Variable	*df* (error *df*)	*F*	*p*	η_p^2
Intercept	1 (71)	45.973	<.001	.393
Exerciser sex	1 (71)	55.223	<.001	.438
Anaerobic fitness	1 (71)	3.917	.052	.052
Condition	1 (71)	0.058	.810	.001
Exercise bout	2.094 (148.665)	0.229	.805*	.003
Exercise bout × condition	2.094 (148.665)	5.344	.005*	.070

*Greenhouse-Geisser interpretation of *F* due to violation of assumption of sphericity.

Table S2.2 Descriptive statistics and post-hoc contrast summaries for main mixed ANOVA on total anaerobic outputs (related to Table S2.1)

	**Bout 1**		**Bout 2**		**Bout 3**		**Bout 4**	
	**Estimated Marginal Mean**	***SD***	**Estimated Marginal Mean**	***SD***	**Estimated Marginal Mean**	***SD***	**Estimated Marginal Mean**	***SD***
Solo Condition	9754.06	2794.94	8850.40	2612.32	8499.41	2573.17	8043.00	2368.51
Companion Condition	10420.75	3113.74	9196.38	2295.19	8265.79	2127.69	8124.23	1957.74

Post-hoc Contrasts

**Contrast**	***df*** **(error** ***df*****)**	***F***	***p***	η_p^2
Bout 1 – Bout 2	1 (71)	2.270	.136	.176
Bout 1 – Bout 3	1 (71)	8.732	.004	.331
Bout 1 – Bout 4	1 (71)	4.714	.033	.250

Table S3.1 Results of mixed ANOVA on peak power outputs (see also Table S3.2)

**Variable**	***df* (error *df*)**	***F***	***p***	η_p^2
Intercept	1 (71)	48.439	<.001	.406
Exerciser sex	1 (71)	37.057	<.001	.343
Anaerobic fitness	1 (71)	2.237	.139	.031
Condition	1 (71)	0.057	.812	.001
Exercise bout	2.465 (175.022)	0.460	.672*	.006
Exercise bout × condition	2.465 (175.022)	4.357	.009*	.058

*Greenhouse-Geisser interpretation of *F* due to violation of assumption of sphericity.

Table S3.2 Descriptive statistics and post-hoc contrast summaries for mixed ANOVA on peak power outputs (related to Table S3.1)

	Bout 1		Bout 2		Bout 3		Bout 4	
	**Estimated Marginal Mean**	***SD***	**Estimated Marginal Mean**	***SD***	**Estimated Marginal Mean**	***SD***	**Estimated Marginal Mean**	***SD***
Solo Condition	432.31	131.98	405.75	126.80	395.47	118.39	393.84	88.08
Companion Condition	458.25	131.91	429.17	106.40	394.19	94.59	383.10	105.29

Post-hoc Contrasts

Contrast	*df* (error *df*)	*F*	*p*	η_p^2
Bout 1 – Bout 2	1 (71)	0.193	.661	.003
Bout 1 – Bout 3	1 (71)	4.673	.034	.062
Bout 1 – Bout 4	1 (71)	6.618	.012	.085

Table S4 Results of mixed ANOVA on average heart rate

Variable	*df* (error *df*)	*F*	*p*	η_p^2
Intercept	1 (60)	504.465	<.001	.894
Exerciser sex	1 (60)	1.460	.232	.024
Anaerobic fitness	1 (60)	1.042	.312	.017
Condition	1 (60)	0.137	.712	.002
Time	1.370 (82.217)	1.783	.184*	.029
Time × condition	1.383 (82.217)	1.843	.175*	.030

This analysis included 31 exercisers from the solo condition and 33 exercisers from the companion condition (the heart rate sensor failed to yield complete data for the remaining 11 exercisers, so their data was not included in this analysis).
Average heart rates were calculated for exercise bouts 1, 2, 3, and 4.
*Greenhouse-Geisser interpretation of *F* due to violation of assumption of sphericity.

Table S5.1 Results of mixed ANOVA on rates of perceived exertion (see also Table S5.2)

Variable	*df* (error *df*)	*F*	*p*	η_p^2
Intercept	1 (73)	2556.272	<.001	.972
Condition	1 (73)	0.001	.977	<.001
Exercise bout	1.912 (139.583)	19.606	<.001*	.212
Exercise bout × condition	1.912 (139.583)	5.343	.007*	.068

*Greenhouse-Geisser interpretation of *F* due to violation of assumption of sphericity.

Table S5.2 Descriptive statistics and post-hoc contrast summaries for mixed ANOVA on rates of perceived exertion (related to Table S5.1)

	**Bout 1**		**Bout 2**		**Bout 3**		**Bout 4**	
	**Estimated Marginal Mean**	***SD***	**Estimated Marginal Mean**	***SD***	**Estimated Marginal Mean**	***SD***	**Estimated Marginal Mean**	***SD***
Solo Condition	11.351	2.111	11.784	2.057	12.676	1.796	13.054	1.929
Companion Condition	11.947	2.640	12.132	2.517	12.289	2.492	12.553	2.435

Post-hoc Contrasts

**Contrast**	***df* (error *df*)**	***F***	***p***	η_p^2
Bout 1 – Bout 2	1 (73)	4.966	.029	.064
Bout 1 – Bout 3	1 (73)	21.271	<.001	.226
Bout 1 – Bout 4	1 (73)	26.721	<.001	.268

Table S6 Results of mixed ANOVA on reports of physical discomfort

**Variable**	***df* (error *df*)**	***F***	***p***	η_p^2
Intercept	1 (73)	342.431	<.001	.824
Condition	1 (73)	1.612	.208	.022
Exercise bout	1.784 (130.241)	31.854	<.001	.304
Exercise bout × condition	1.935 (130.241)	0.536	.566	.007

Table S7 Mixed ANOVA on total anaerobic outputs with the exerciser-companion relationship component as moderator of exercise bout × condition interaction

**Variable**	***df* (error *df*)**	***F***	***p***	η_p^2
Intercept	1 (45)	24.365	<.001	.351
Exerciser sex	1 (45)	28.623	<.001	.389
Anaerobic fitness	1 (45)	3.674	.062	.075
Condition	1 (45)	0.025	.876	.001
Exercise bout	2.308 (103.869)	0.103	.904*	.003
Exercise bout × condition × exerciser-companion relationship component	2.308 (103.869)	2.294	.098*	.049

*Greenhouse-Geisser interpretation of *F* due to violation of assumption of sphericity.

Table S8 Mixed ANOVA on total anaerobic outputs with perceived daily social support as moderator of exercise bout × condition interaction

**Variable**	***df* (error *df*)**	***F***	***p***	η_p^2
Intercept	1 (40)	23.803	<.001	.373
Exerciser sex	1 (40)	25.421	<.001	.389
Anaerobic fitness	1 (40)	2.400	.129	.057
Condition	1 (40)	0.974	.330	.024
Exercise bout	2.277 (91.092)	0.611	.565*	.015
Exercise bout × condition × support in daily life	2.277 (91.092)	.522	.619*	.013

*Greenhouse-Geisser interpretation of *F* due to violation of assumption of sphericity.

Table S9 Mixed ANOVA on total anaerobic outputs with need for social assurance as moderator of exercise bout × condition interaction

**Variable**	***df* (error *df*)**	***F***	***p***	η_p^2
Intercept	1 (46)	21.855	<.001	.322
Exerciser sex	1 (46)	31.893	<.001	.409
Anaerobic fitness	1 (46)	2.688	.108	.055
Condition	1 (46)	<0.001	.996	.000
Exercise bout	2.029 (93.355)	0.329	.805*	.007
Exercise bout × condition × need for social assurance	2.029 (93.355)	0.163	.853*	.004

*Greenhouse-Geisser interpretation of *F* due to violation of assumption of sphericity.

Table S10.1 Mixed ANOVA on total anaerobic outputs with neuroticism as a moderator of the exercise bout × condition interaction (see also Table S10.2)

**Variable**	***df* (error *df*)**	***F***	***p***	η_p^2
Intercept	1 (46)	44.495	<.001	.514
Exerciser sex	1 (46)	26.237	<.001	.385
Anaerobic fitness	1 (46)	0.031	.860	.001
Condition	1 (46)	0.201	.656	.005
Exercise bout	1.983 (83.274)	0.082	.920*	.002
Exercise bout × condition × neuroticism	1.983 (83.274)	5.693	.005*	.119

*Greenhouse-Geisser interpretation of *F* due to violation of assumption of sphericity.

Table S10.2 Descriptive statistics and post-hoc contrast summaries for mixed ANOVA on total anaerobic outputs with neuroticism as a moderator of the exercise bout × condition interaction (related to Table S10.1)

		Bout 1		Bout 2		Bout 3		Bout 4	
		**Estimated Marginal Mean**	***SE***	**Estimated Marginal Mean**	***SE***	**Estimated Marginal Mean**	***SE***	**Estimated Marginal Mean**	***SE***
Low Neuroticism	Solo Condition	9372.73	637.32	8168.46	576.17	8220.84	534.58	8011.03	514.34
	Companion Condition	11992.16	648.80	9903.40	586.55	8844.47	544.21	8669.18	523.60
High Neuroticism	Solo Condition	10376.46	529.01	9320.39	478.25	8915.51	443.73	8337.81	426.93
	Companion Condition	9319.53	503.55	8426.38	455.24	7752.37	422.38	7553.42	406.38

Post-hoc Contrasts

Contrast	*df* (error *df*)	F	p	η_p^2
Bout 1 – Bout 2	1 (46)	2.691	.108	.060
Bout 1 – Bout 3	1 (46)	8.312	.006	.165
Bout 1 – Bout 4	1 (46)	8.038	.007	.161

Table S11 Robust two-way mixed ANOVA on rates of perceived exertion

Variable	Q	p
Condition	0.112	.740
Exercise bout	13.149	<.001
Exercise bout × condition	3.894	.016

Figures

Fig. S1 Mean (± 1 SE) physical discomfort by condition

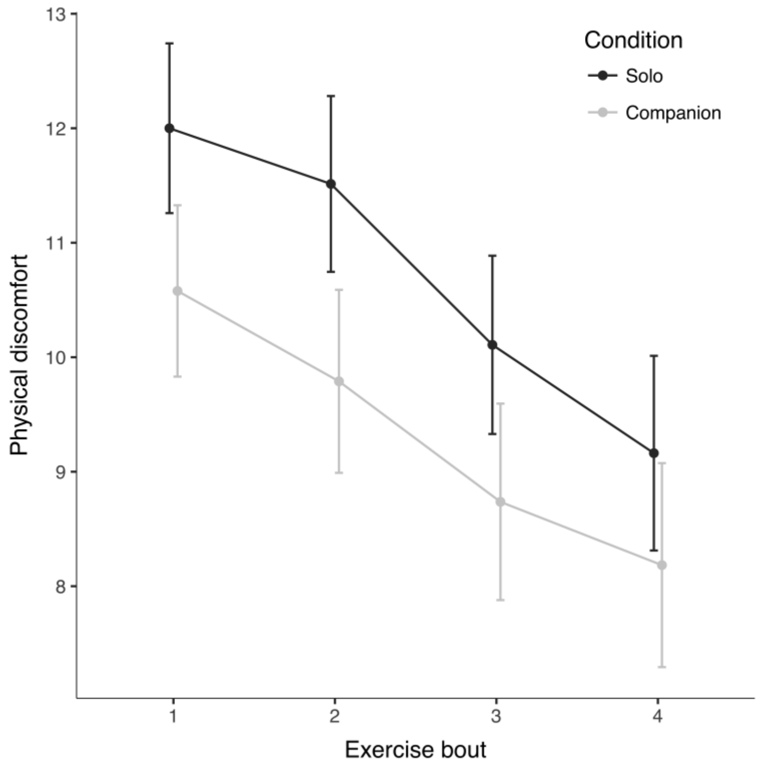

Fig. S2.1 Normal Q-Q plot of total anaerobic outputs for exercise bout 2 in the solo condition

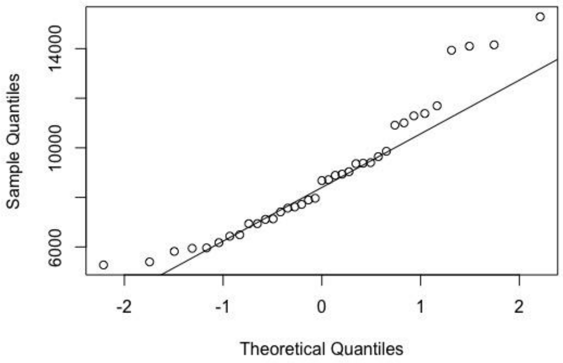

Fig. S2.2 Normal Q-Q plot of total anaerobic outputs for exercise bout 3 in the solo condition

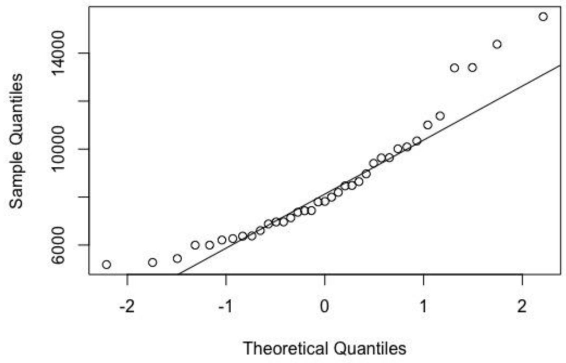

Fig. S2.3 Normal Q-Q plot of total anaerobic outputs for exercise bout 4 in the solo condition

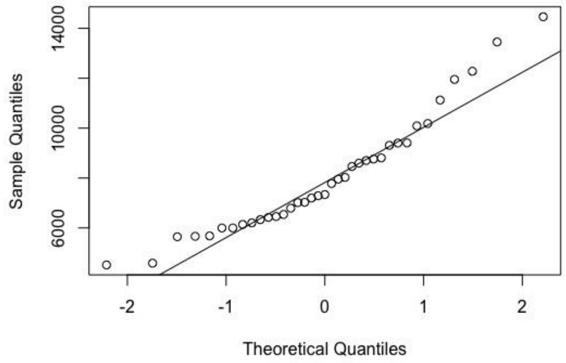

Fig. S3.1 Normal Q-Q plot of physical discomfort for exercise bout 4 in the solo condition

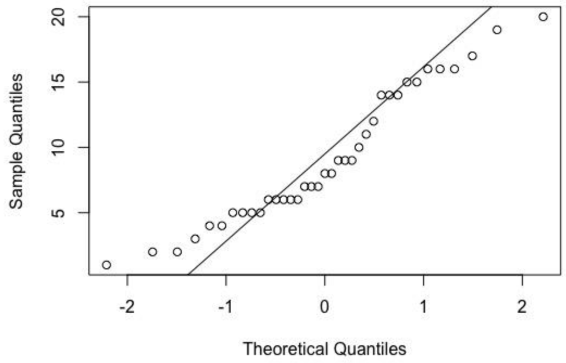

Fig. S3.2 Normal Q-Q plot of physical discomfort for exercise bout 3 in the companion condition

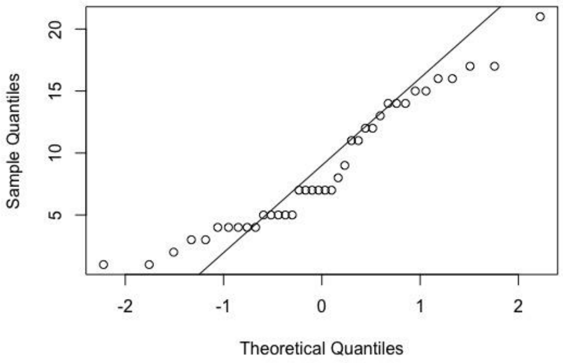

Fig. S3.3 Normal Q-Q plot of physical discomfort for exercise bout 4 in the companion condition

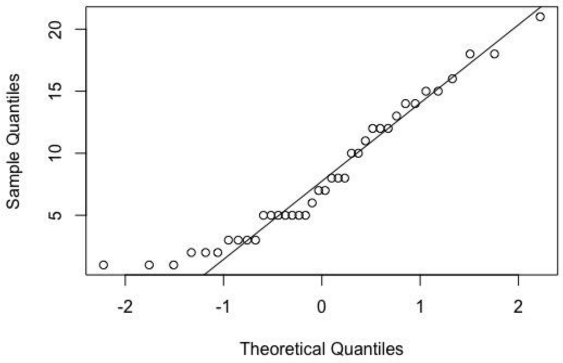

Fig. S4.1 Normal Q-Q plot of rates of perceived exertion for exercise bout 1 in the solo condition

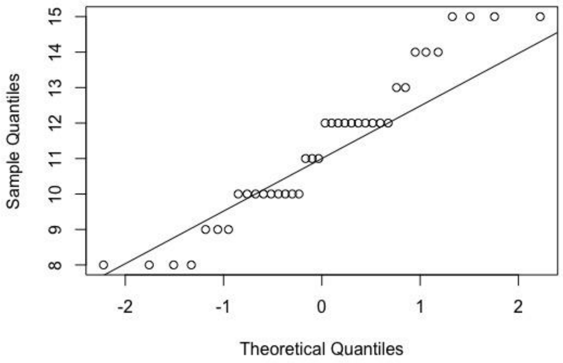

Fig. S4.2 Normal Q-Q plot of rates of perceived exertion for exercise bout 2 in the solo condition

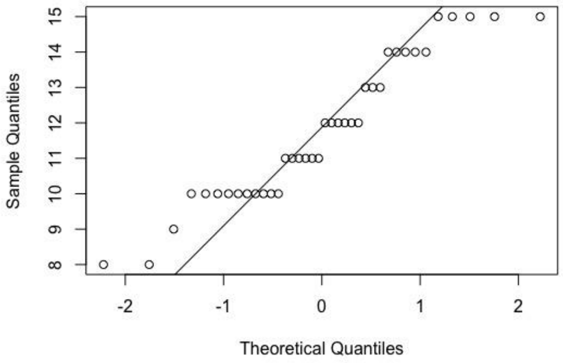

Fig. S4.3 Normal Q-Q plot of rates of perceived exertion for exercise bout 3 in the solo condition

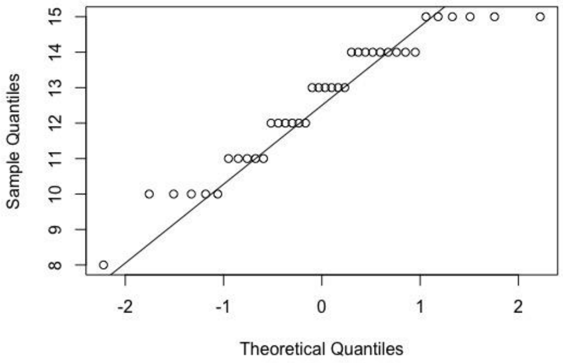

Fig. S4.4 Normal Q-Q plot of rates of perceived exertion for exercise bout 4 in the solo condition

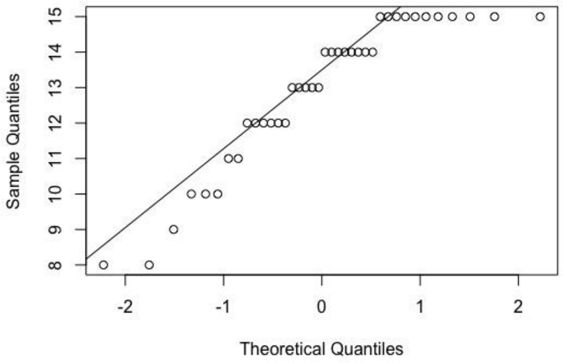

Fig S4.5 Normal Q-Q plot of rates of perceived exertion for exercise bout 1 in the companion condition

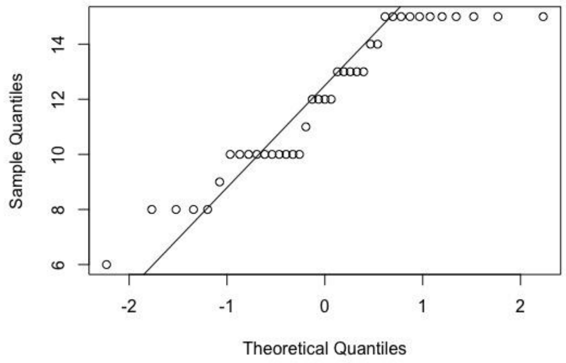

Fig. S4.6 Normal Q-Q plot of rates of perceived exertion for exercise bout 2 in the companion condition

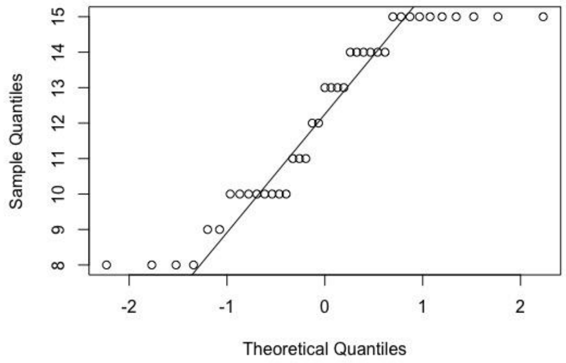

Fig. S4.7 Normal Q-Q plot of rates of perceived exertion for exercise bout 3 in the companion condition

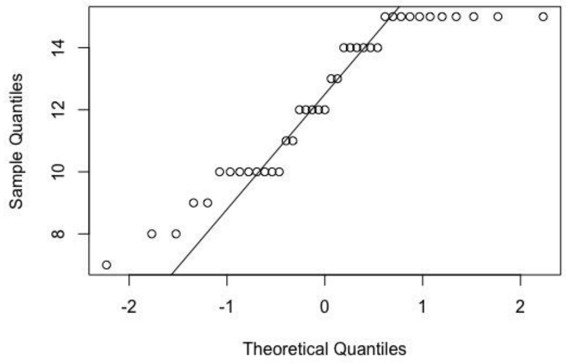

Fig. S4.8 Normal Q-Q plot of rates of perceived exertion for exercise bout 4 in the companion condition

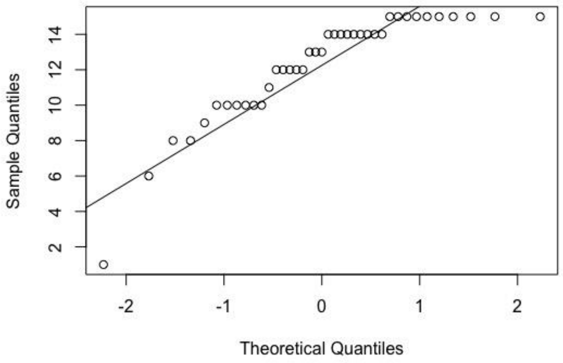

Fig. S5 Variance of rates of perceived exertion across exercise bouts in the solo (*a*) and companion (*b*) conditions

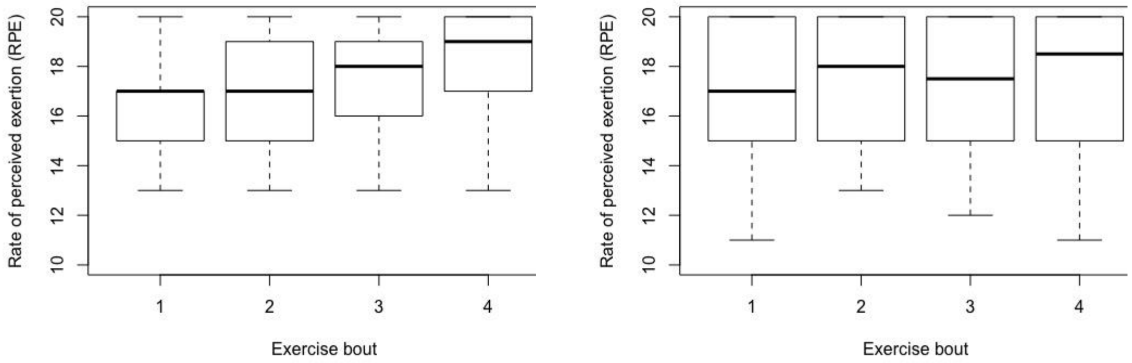

References

Baecke, J., Burema, J., & Frijters, J. (1982). A short questionnaire for the measurement of habitual physical activity in epidemiological studies. *The American Journal of Clinical Nutrition, 36*(5), 936-942.

Borg, G. A. (1982). Psychophysical bases of perceived exertion. *Medicine & Science in Sports & Exercise, 14*(5), 377-381.

Box, G. E. (1949). A general distribution theory for a class of likelihood criteria. *Biometrika, 36*(3/4), 317-346.

Eisenberger, N. I., Master, S. L., Inagaki, T. K., Taylor, S. E., Shirinyan, D., Lieberman, M. D., & Naliboff, B. D. (2011). Attachment figures activate a safety signal-related neural region and reduce pain experience. *Proceedings of the National Academy of Sciences of the United States of America, 108*(28), 11721-11726. doi:10.1073/pnas.1108239108

Field, A., Miles, J., & Field, Z. (2012). *Discovering Statistics Using R*. London: Sage.

Glass, G. V., Peckham, P. D., & Sanders, J. R. (1972). Consequences of Failure to Meet Assumptions Underlying the Fixed Effects Analyses of Variance and Covariance. *Review of Educational Research, 42*(3), 237-288. doi:10.2307/1169991

Lee, R. M., & Robbins, S. B. (1995). Measuring belongingness: The Social Connectedness and the Social Assurance Scales. *Journal of Counseling Psychology, 42*(2), 232-241.

McArdle, W. D., Katch, F. I., & Katch, V. L. (2010). *Exercise Physiology: Nutrition, Energy, and Human Performance*. Philadelphia: Lippincott Williams & Wilkins.

Morrison, D. F. (1998). *Multivariate analysis, overview*: Wiley Online Library.

Rammstedt, B., & John, O. P. (2007). Measuring personality in one minute or less: A 10-item short version of the Big Five Inventory in English and German. *Journal of Research in Personality, 41*(1), 203-212. doi:10.1016/j.jrp.2006.02.001

Wilcox, R. R. (2012). *Introduction to Robust Estimation and Hypothesis Testing*. Cambridge, MA: Academic Press.

Zimet, G. D., Dahlem, N. W., Zimet, S. G., & Farley, G. K. (1988). The Multidimensional Scale of Perceived Social Support. *Journal of Personality Assessment, 52*(1), 30-41.
